# Multi-omics analysis reveals the potential for fermented *Cordyceps militaris* mushroom substrate in laying hens

**DOI:** 10.3389/fmicb.2026.1807060

**Published:** 2026-06-17

**Authors:** Hailong Zhang, Lili Ma, Li Jia, Yujing Li, Yuxin Wang, Wenwen Wang, Wenting Wu, Hai Wang, Hongjie Li, Yaxiong Zhang, Guoshun Chen, Ke Hou, Jinjie Dong

**Affiliations:** 1 College of Forestry Engineering, Gansu Forestry Voctech University, Tianshui, China; 2 College of Animal Science and Technology, Gansu Agricultural University, Lanzhou, China

**Keywords:** laying hens, metabolomics, metagenomics, production performance, quorum sensing

## Abstract

This study examines how varying levels of fermented *Cordyceps militaris* mushroom substrate (CMMS) in laying hen diets affect production performance, digestive health, immunity, cecal microbiota, metabolites, and quorum-sensing functions. Fermentation reduced CMMS dry matter, NDF, and phosphorus content (*p* < 0.05). Replacing 30% of the diet with fermented CMMS significantly improved laying rate, egg weight, feed intake, and feed efficiency (*p* < 0.05), while enhancing yolk color, Haugh units, and lipase activity. A 20% substitution increased nutrient digestibility and immunoglobulin levels (*p* < 0.05). Metagenomic analysis revealed increased abundance of Phocaeicola, Alistipes, and Parabacteroides (*p* < 0.05) with enhanced energy metabolism and specific gene families. Metabolomic analysis identified 1,529 differentially expressed metabolites, with carboxylic acids being most prevalent (21.20%), and enhanced taurine/hypotaurine metabolism and GPI-anchor biosynthesis. Parabacteroides showed negative correlations with certain metabolites, while Alistipes correlated positively with PemK/MazF family genes (*p* < 0.001). CMMS fermented feed proportions influence cecal microbiota, their metabolites, and quorum sensing in laying hens, affecting production, digestibility, immunity, metabolism, and health, demonstrating CMMS potential as alternative poultry nutrition.

## Introduction

Chickens are one of the most widely farmed animals in the world, providing humans with large quantities of meat and eggs. It is estimated that the global chicken population exceeded 60 billion in 2021 (Feng et al., 2021). The gastrointestinal tract of chickens is home to a large number of microorganisms, and their importance to the health and metabolism of the host has been recognized (Bindari and Gerber, 2022). The majority of these microbial organisms are bacterial species, which fall into three main categories: symbiotic bacteria, pathogenic bacteria, and “probiotics.” The structural traits and social habits of these microorganisms are influenced by a variety of factors, including their hereditary makeup, the age of the organism they inhabit, their surroundings, the food they consume, and the addition of dietary supplements (Aruwa et al., 2021; Dai et al., 2022; Pandit et al., 2018). Notably, alterations in these microecological elements have been confirmed to exert a direct impact on the health status and production traits of poultry (Baxter et al., 2021; Sasaki et al., 2014; Ndlebe et al., 2023), indicating that a distinct correlation exists between intestinal microbiota and the health and production performance of poultry. As an illustration, relevant research has revealed that broiler chickens with high feed conversion ratios (FCR) and those with low FCR exhibit differences in bacterial abundance (Stanley et al., 2014). Production performance-related parameters including breeding density, as well as the protein and energy percentages in the diet are capable of regulating key microbiome components involved in nutritional processes and metabolic activities. In turn, this regulation affects the growth of the host and the efficiency of feed utilization (McKenna et al., 2020). Therefore, gaining a comprehensive understanding of the intestinal microbiome in chickens is of great significance for enhancing their health level and production capacity. Studies indicate that incorporating probiotics into one’s diet can significantly influence the makeup of gut bacteria, thereby decreasing susceptibility to disease. Additionally, the inclusion of plant-based feed components has demonstrated the ability to shape the intestinal microbial community, ultimately enhancing poultry health (Sayed et al., 2023; Joo et al., 2022; Shi et al., 2020; Lee et al., 2020; Chen et al., 2019).

Nevertheless, prior to optimizing the intestinal microbiota of chickens, a thorough comprehension of the gut microbiota itself and the interactions among its constituent members is essential. Quorum-sensing (QS) systems refer to the biological process where bacteria pump out signaling molecules that enable them to gauge the local population density and regulate specific gene expression accordingly (Federle and Bassler, 2003). Numerous physiological and biochemical processes of bacteria, including bioluminescence emission, biofilm formation, bacterial pathogenicity, spore creation, conjugation processes, and bacteriocin synthesis, have been shown to be modulated by the QS phenomenon (Lowery et al., 2008). Relevant research has also indicated that autoinducer-2 (AI-2) plays a pivotal role in mediating intercellular communication within microbial communities, and this signaling molecule is extensively employed by both gram-positive and gram-negative bacterial species (Xu and Liu, 2011). Previous research has created a metagenomic dataset of the intestinal microbiota of chickens and demonstrated how some drugs affect chicken growth (Foley and Grant, 2007; Gilroy et al., 2021). Moreover, the gut flora in chickens is incredibly diverse and influenced by their location, stage in life, gender, and how they are nourished, all factors that play a significant role in the chickens’ well-being and their efficiency as producers (Rychlik, 2020). Yet, we are still in the dark about how the gut bacteria of chickens on various diets vary in terms of their makeup and how they work. Plus, there’s a whole lot less insight into what types of bugs and functions are lurking in hens’ digestive tracts.

The shortage and rising prices of conventional feed resources are one of the challenges facing the livestock industry (Li, 2023). Therefore, the development of unconventional feed has become an interesting topic in livestock research (Santoso and Setiadi, 2016), including the distillers’ grains (Li et al., 2025b), straw (Li et al., 2025b), and cottonseed meals (Zhang et al., 2024). However, these unconventional feed resources have certain disadvantages, such as high crude fiber content and the presence of antinutritional factors (Natesh et al., 2017; Cheng et al., 2022).

*Cordyceps militaris* (CM) stands out as a consumable fungus that falls within the Cordycepitaceae family, and it’s been a staple in traditional medicine for ages, thanks to its impressive nutritional and medicinal properties (Liu et al., 2016; Das et al., 2010). CMMS is a by-product of CM cultivation, mainly composed of CM mycelium and fermented wheat (Guo et al., 2010), and is a high-quality, renewable, unconventional feed. However, whether CMMS can be used as a feed resource in egg production and its mechanism of action remains unclear. Hence, the investigation delved into the alterations in the gut bacteria composition, specific functions, and resulting metabolites within Hy-Line Brown laying hens, using various concentrations of fermented CMMS to replace a portion of their feed. Additionally, it looked at how this affected digestive enzyme activity, digestibility, and immune responses. This research aimed to discern how these alterations could influence the laying hens’ productivity. The key goals of the study were to: (1) to reveal the changes in the cecal microbiota, some of its functions, and metabolic products of laying hens under a diet partially replaced with fermented CMMS; (2) to investigate the effects of blind intestine microbial communities, their QS functions, and metabolic products on production performance under the influence of fermented *Cordyceps militaris* waste mushroom substrate; and (3) to analyze the relationship between microbial communities and their QS functions and metabolic products. The above results will provide a reference for the feed utilization of fermented *Cordyceps militaris* waste mushroom substrates in laying hens.

## Methods

### Experimental design and sample collection

For this investigation, we chose 144 Hy-Line Brown hens, all about 18 weeks old and in the laying stage. They were all healthy and started producing eggs. These birds were free to feast and quench their thirst during the trial, all under the same, strict feeding and care routine. The hens came from a single source—Jia Yuan Poultry Breeding Technology Demonstration Park in Lintao County, Gansu Province, China—to keep any potential discrepancies to a minimum. The chicks were shuffled into four different groups, each with six pens holding six birds each. Here’s the lowdown on the diet they were given (refer to Supplementary Table S1): one group received the standard feed (AMC), while the others saw different percentages of their standard feed swapped out for fermented CMMS. Specifically, one group got 10% fermented CMMS (BMC), another got 20% (CMC), and the last group got a heftier 30% (DMC) mixed in with their regular grub. CMMS is sourced from Zhongxing Mushroom Industry Co., Ltd. (Tianshui City, Gansu Province, China). Fermented CMMS was prepared between July and August 2024 (successfully prepared as indicated by the distinct release of a wine-like aroma). CMMS consists of circular mushroom substrate cakes measuring 5 cm in diameter and 2 cm in thickness, with a moisture content of 65–70%. The entire mushroom substrate was cut into 2–5 cm chunks and placed into blue cylindrical polyethylene silage bags. Anaerobic fermentation proceeded for 60 days at room temperature (15–26 °C). Sample collection was completed in mid-May 2025. During production performance testing, the total egg weight and feed intake for each pens were recorded over three consecutive days in the middle of the trial. On the final day of feed intake measurement, one egg was randomly collected from each pens for egg quality testing. For digestibility analysis, feed and feces samples were collected from each pens over three consecutive days following the production performance and egg quality measurements. Immunoglobulins were measured using blood samples collected at slaughter. Enzyme activity, metagenomics, and metabolomics were measured using cecal samples collected post-slaughter. One hen was randomly selected from each pens for euthanasia. After bleeding and euthanizing the experimental chickens, their body surfaces were disinfected with 75% ethanol. Next, the cecal tissue was rapidly excised and securely ligated with sterile cotton thread at its distal end. The resected intestinal material and anaerobic culture packets were then transferred to airtight pouches, properly sealed, and kept chilled at 4 °C in preparation for microbial isolation. Concurrently, the remaining specimens were flash-frozen in liquid nitrogen, transported under cold chain conditions to the research facility, and archived in a − 80 °C freezer subsequent to genomic analysis.

### Nutritional components of fermented CMMS: measurement of laying hen production performance, immune indices, and physiological indices

The nutritional components, both before and after fungal bran fermentation, and apparent digestibility (using the acid insoluble ash, AIA method. Digestibility of the nutrient of interest = 100 − (A1/A2 × N2/N1) × 100% Where: N1: Content of the nutrient of interest in the feed (%); N2: Content of the nutrient of interest in the fecal sample (%); A1: Acid-insoluble ash content in the feed (%); A2: Acid-insoluble ash content in the fecal sample (%).) were determined using methods from the literature (Horvath et al., 2021). Throughout the experimental period, researchers tracked daily egg weight and feed consumption across three consecutive days, then computed the production rate and feed efficiency ratio. In addition, egg samples were gathered for comprehensive quality assessment (Kim et al., 2025). Enzyme activity in the cecum of laying hens was measured using the Lipase ELISA kit (Gansu Aokexin Biotechnology Co., Ltd., Gansu, China), Trypsin ELISA kit (Gansu Aokexin Biotechnology Co., Ltd., Gansu, China), and Alpha-amylase ELISA kit (Gansu Aokexin Biotechnology Co., Ltd., Gansu, China). The IgA ELISA kit (Gansu Aokexin Biotechnology Co., Ltd., Gansu, China), IgM ELISA kit (Gansu Aokexin Biotechnology Co., Ltd., Gansu, China), and IgG ELISA kit (Gansu Aokexin Biotechnology Co., Ltd., Gansu, China) were used to determine the immunoglobulin content in the serum of the laying hens.

### DNA extraction, library preparation, and sequencing

For isolating genomic DNA from our collected samples, we utilized one of two MAGEN extraction kits—the MagPure Stool DNA KF Kit B or the Magnetic Bead Fecal and Soil Genome Extraction Kit—both hailing from Guangzhou, China. In each case, we meticulously adhered to the protocols supplied by the manufacturer. Each sample’s 100–200 milligram portion was transferred to a grinding bead-filled centrifuge tube to initiate the process. Next, added 1 mL of buffer ATL/PVP-10 to the tube before putting it through the wringer with a grinder from Shanghai Jingxin Tech back in China. Once that was done, the sample sat tight under specific conditions and then got spun around at 14000 × g for 5 min in an Eppendorf centrifuge fresh from Germany. The clear liquid supernatant was skillfully pipetted into a fresh, sterile centrifuge container. Following the initial step, a generous 0.6 mL of PCI buffer was blended into the supernatant, followed by a quick but vigorous 15-s whirl to ensure thorough mixing. After spinning at 18,213 g for a full 10 min, the clear liquid portion was meticulously transferred to a deep-well plate that had already been treated with a magnetic bead binding solution. This concoction included 600 μL of magnetic bead buffer, a dash of 20 μL Proteinase K, a pinch of 5 μL RNase A, a total of 700 μL for each of the three Wash Buffers (numbers 1, 2, and 3), and a final 100 μL of Elution Buffer. The plate was then placed in its designated spot in the automated DNA extraction system, the Kingfisher from Thermo Fisher, United States, and the extraction protocol was set into motion. Post-run, the extracted DNA was meticulously transferred into a 1.5 mL microcentrifuge tube and was stored neatly, adhering to the appropriate storage parameters to maintain its integrity.

We built our libraries using either the MGIEasy Universal DNA Library Prep Set or the BGI Optimal DNA Library Prep Kit, both from MGI-Shenzhen and BGI-Shenzhen respectively, following established protocols. To kick things off, we measured out the right amount of genomic DNA and put it through mechanical fragmentation to get DNA pieces of the desired length. After breaking up the DNA, we used magnetic bead technology to cherry-pick fragments that hit the size sweet spot. Next, these carefully selected fragments underwent end-repair treatment to give them blunt ends. We then tacked on a single “A” nucleotide to the 3′ end of these blunt fragments via A-tailing. Following that, we attached specialized adapters to both ends of our modified DNA pieces using a ligation reaction. In the final stretch, we amplified these adapter-bound fragments through PCR and put them through the wringer with quality control checks to make sure our libraries were shipshape.

After getting the final double-stranded library products, they were heated up to break them apart into single-stranded molecules. Then came the circularization step, where the single-stranded DNA was coaxed into forming loops. Before moving forward, any leftover linear DNA fragments were chewed up and removed to ensure only the circular stuff remained. The next phase involved rolling circle amplification with phi29 DNA polymerase, which turned each original single-stranded molecule into DNA nano balls packing about 300 copies apiece. These little balls were then placed onto the nanoarray platform, where the sequencer cranked out paired-end reads 100 or 150 bases long—all thanks to the DNBSEQ-G400/T7/T10 system made by BGI in Shenzhen, China.

### Metagenomic data analysis methods

Before diving into the analysis, we first put all raw sequencing data through its paces by trimming low-quality bases using SOAPnuke version 1.5.2. Next, we ran the trimmed sequences through SOAP2 to map them against the host genome, which allowed us to weed out any host-derived reads—though this step was only relevant for samples that originated from the host. After filtering out the low-quality reads, we then turned to MEGAHIT to assemble the remaining high-quality sequences *de novo*. Once assembly was complete, we tossed out any contigs that were under 200 bp in length to clean up the dataset. Finally, we employed MetaGeneMark to predict genes on the remaining, more substantial contigs for our downstream analyses. CD-HIT eliminated redundant gene sequences at 95% identity and 90% coverage thresholds. The Salmon tool was utilized for gene quantification, and a gene abundance matrix was constructed accordingly. The protein sequences encoded by the predicted genes were aligned against functional databases (KEGG and Swiss-Prot) using DIAMOND software, with an E-value cutoff of 1e−5 to generate functional annotation information. Taxonomic annotations were assigned based on the Kraken LCA algorithm. To pinpoint taxonomic and functional characteristics (including Genera, Phyla, and KOs) that showed statistically significant variations across the studied groups, we utilized the Wilcoxon rank-sum test to analyze their respective abundance profiles. Differentially enriched KEGG pathways were detected according to reporter scores, with an absolute reporter score of 1.65 or higher set as the threshold for statistical significance. To assess ecological variation, we employed the Bray–Curtis dissimilarity index, followed by a principal coordinate analysis (PCoA) to visualize patterns in community composition. All statistical evaluations were carried out using R, specifically applying the Wilcoxon rank-sum test and Kruskal-Wallis H test for comparative analyses. Following normalization of species abundance data, we utilized the Kruskal-Wallis rank sum test, complemented by linear discriminant analysis (LDA) to identify potential biomarker species across various fermented substrate ratios (*p* < 0.05, LDA score > 4). For additional insights, nonparametric factor analysis incorporating both Kruskal-Wallis tests and rank-based evaluations was conducted, with the Python LEfse package facilitating the detection of significant variations in species abundance profiles.

### Metabolomics measurement

The frozen specimens (cecal chyme) were brought back to room temperature incrementally at 4 °C, with each target sample carefully portioned out at 50–100 mg and placed into centrifuge containers. Each tube then received 0.2 mL of ice-cold ultrapure water before being thoroughly mixed for a full minute. Following this, 0.8 mL of a prechilled extraction liquid—comprising a 1:1 (v/v) blend of methanol and acetonitrile—was introduced to the mix. An additional minute of homogenization preceded a 30-min ultrasonic extraction period conducted under cold conditions. Protein precipitation was achieved by letting the samples sit undisturbed at −20 °C for an hour, after which they were spun in a centrifuge at 12000 rpm and 4 °C for 10 min. The supernatant was subsequently dried completely via vacuum freeze-drying. The remaining pellets were brought back into solution by adding 0.2 mL of a 30% acetonitrile mixture, thoroughly mixed again, and then subjected to centrifugation at 14000 rpm and 4 °C for a quarter of an hour. The upper liquid layer was carefully collected and deemed ready for examination using analytical instrumentation.

The samples were put through both a deep dive into their qualitative and a meticulous quantitative examination via a UPLC-Orbitrap-MS setup. This system was a powerhouse, combining a Vanquish UPLC system with an HFX mass spectrometer. Here’s how we cranked up the details: For the UPLC part of the process, we went with a Waters HSS T3 column—measuring 100 × 2.1 mm with 1.8 μm particles. We kept the column nice and cool at a steady 40 degrees celsius. The mobile phase flowed at a rate of 0.3 mL/min, with a 2 μL sample injected into the system. The solvent mixture consisted of two components: mobile phase A, which was essentially Milli-Q water with a 0.1% formic acid kick, and mobile phase B, acetonitrile also containing 0.1% formic acid. The gradient program followed this exact itinerary: at 0 min, the ratio was 100:0 (A/B, v/v); this composition held steady until the 1-min mark. By 4 min, the mixture had shifted to 40:60 (A/B, v/v). At 6.5 min, it reached 5:95 (A/B, v/v), then immediately jumped back to 100:0 (A/B, v/v) at 6.6 min, maintaining this ratio until the final time point of 8.0 min.

We employed a Q Exactive HFX Hybrid Quadrupole Orbitrap mass spectrometer from Thermo Fisher Scientific to obtain our high-fidelity mass spectrometry data, featuring a heated electrospray ionization (ESI) setup. For data acquisition, we selected the Full-ms-ddMS2 mode. The ESI was operating with the following parameters: sheath gas at 40 arb, auxiliary gas at 10 arb, with spray voltage set to +3,000 V/−2,800 V, source temperature held at 350 °C, and the ion transport tube at 320 °C. For the mass spectrometry scan, we targeted a mass range of 70–1,050 Da (m/z), maintaining a primary resolution of 70,000 and a secondary resolution of 17,500.

We acquired the raw mass spectrometry data utilizing Xcalibur 4.1 software (Thermo Scientific) on the Q-Exactive spectrometer before refining it through Progenesis QI software (Waters Corporation, Milford, United States). The processed data were then seamlessly transferred to Excel format for further examination. Employing the R software suite, we performed multivariate data analysis, incorporating both PCA and OPLS-DA techniques. To assess how each variable contributed to distinguishing between groups, we calculated VIP scores within the OPLS-DA model. We then honed in on metabolites boasting VIP scores exceeding 1, which we subjected to Student’s *t*-test to evaluate univariate statistical disparities in their abundance levels. Our criterion for statistical significance was established at a *p*-value threshold below 0.05.

A principal component analysis, commonly referred to as PCA, was conducted utilizing the statistical functions from the ropls package within the R programming environment. Before diving into unsupervised PCA, we scaled the data to ensure unit variance. Additionally, we conducted partial least squares discriminant analysis (PLS-DA) and orthogonal PLS-DA (OPLS-DA), both via the ropls R package, which provided us with score plots and permutation plots as outcomes. For both PLS-DA and OPLS-DA, we applied Pareto scaling as a preprocessing step. To prevent model overfitting, a permutation test was carried out with 200 iterations. We identified key metabolites—those showing significant variations between groups—by applying two strict criteria: a variable importance in projection (VIP) score of 1 or higher and a statistical significance with a *p*-value of 0.05 or less, with these VIP values being derived directly from the OPLS-DA analysis results. To conduct KEGG annotation and enrichment analysis, we first cross-referenced the identified differential metabolites with the KEGG Compound database^1^ and then mapped these annotated compounds to the KEGG Pathway database.^2^ Finally, we utilized the Chemrich online platform^3^ to perform additional analysis on these metabolites.

### Data analysis

To delve into the statistical intricacies of our study, we employed the sophisticated IBM SPSS Statistics 26.0 software, hailing from the reputable IBM Corp. in Armonk, NY, United States. A Shapiro–Wilk test was performed on all data prior to analysis, and the results indicated that the data followed a normal distribution. We wielded the independent samples *t*-test to suss out any discrepancies in the nutritional profiles of bran samples before and after fermentation. For a more comprehensive look at production prowess and egg quality, we turned to the one-way ANOVA (Post-hoc test using LSD and Duncan’s multiple range test), examining factors like digestibility, enzyme activity, and serum immunoglobulin levels in laying hens. The findings were meticulously presented in the format of “mean ± standard deviation”. To delve deeper, we used Spearman’s correlation analysis to uncover connections between the top 20 bacterial genera and the most pivotal differential metabolites, as well as those with QS genes. We set the bar for statistical relevance at a stringent *p*-value of less than 0.05.

## Results

### Changes in the nutritional components of fermented CMMS and production performance, egg quality, and physical and chemical indicators of laying hens

**Figure 1:**
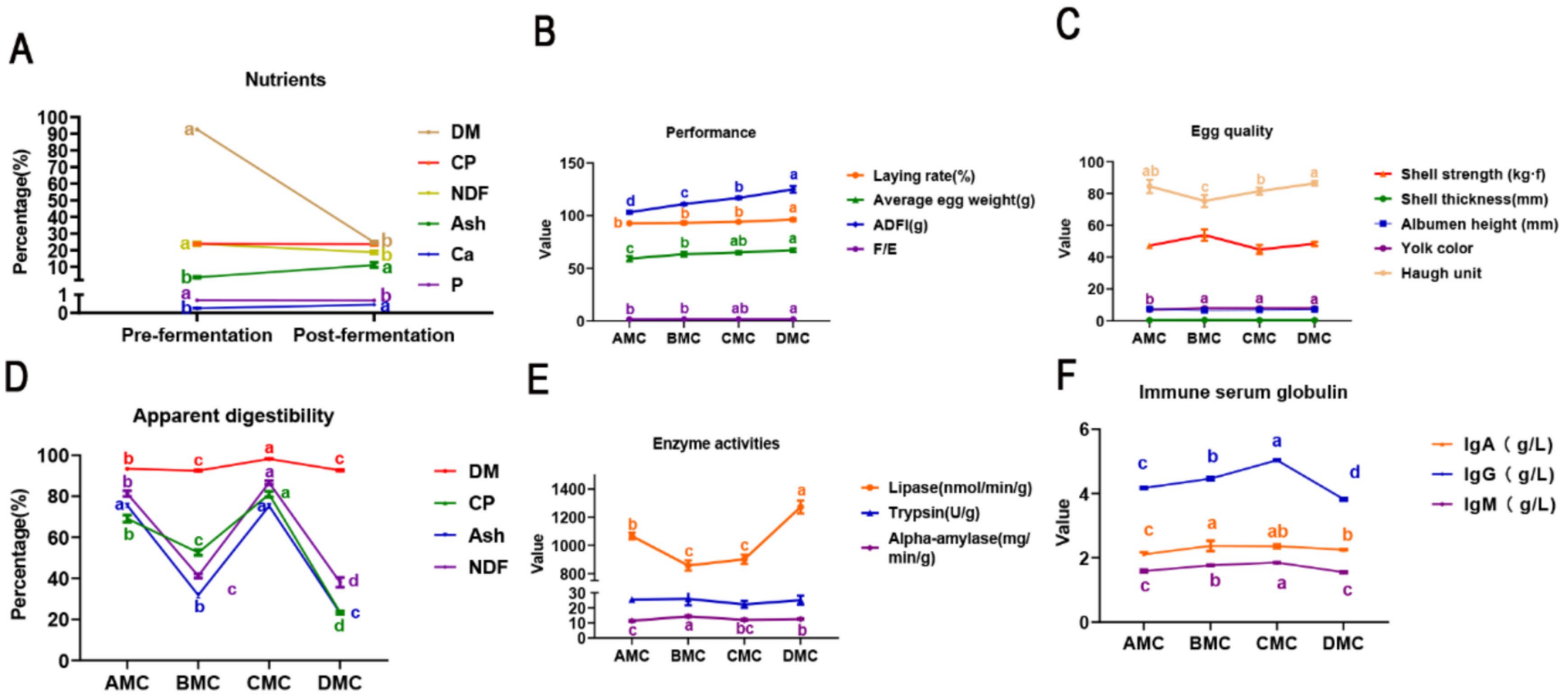
Changes in the nutritional composition of the fermentation substrate for *Cordyceps militaris* mushrooms and analysis of laying hen production performance, egg quality, apparent digestibility, enzyme activity, and immune levels. **(A)** Nutritional indicators. **(B)** Production performance. **(C)** Egg quality. **(D)** Apparent digestibility. **(E)** Enzyme activity. **(F)** Immunoglobulin. Different lowercase letters in the line graph indicate significant differences (*p* < 0.05).

The dry matter, neutral detergent fiber, and phosphorus levels in the fermented CM mycelium were notably reduced (*p* < 0.05). However, there was a significant bump in the crude ash and calcium levels (*p* < 0.05), as depicted in Figure 1A. When it came to egg-laying, the DMC group blew away the competition, boasting significantly higher production rates, bigger egg sizes, more daily feed consumption, and a better feed-to-egg ratio than the other groups (*p* < 0.05), as seen in Figure 1B. In terms of egg quality, the DMC group showed a significant increase in yolk color and Haugh unit (*p* < 0.05) (Figure 1C). In terms of apparent digestibility, the CMC group had significantly higher dry matter, crude protein, and neutral detergent fibers than the other experimental groups (Figure 1D). In terms of cecal enzyme activity, lipase was significantly elevated in the DMC group (*p* < 0.05). In the BMC group, *α*-amylase levels soared, reaching statistical significance (*p* < 0.05) as depicted in Figure 1E. As for the immunoglobulins in the serum, there was a marked increase in IgA and IgM in the CMC group, also reaching statistical significance (*p* < 0.05). Simultaneously, IgG levels in the BMC group experienced a significant rise (*p* < 0.05), as illustrated in Figure 1F.

### Changes in the bacterial community structure of the cecum of laying hens fed different proportions of CMMS

**Figure 2:**
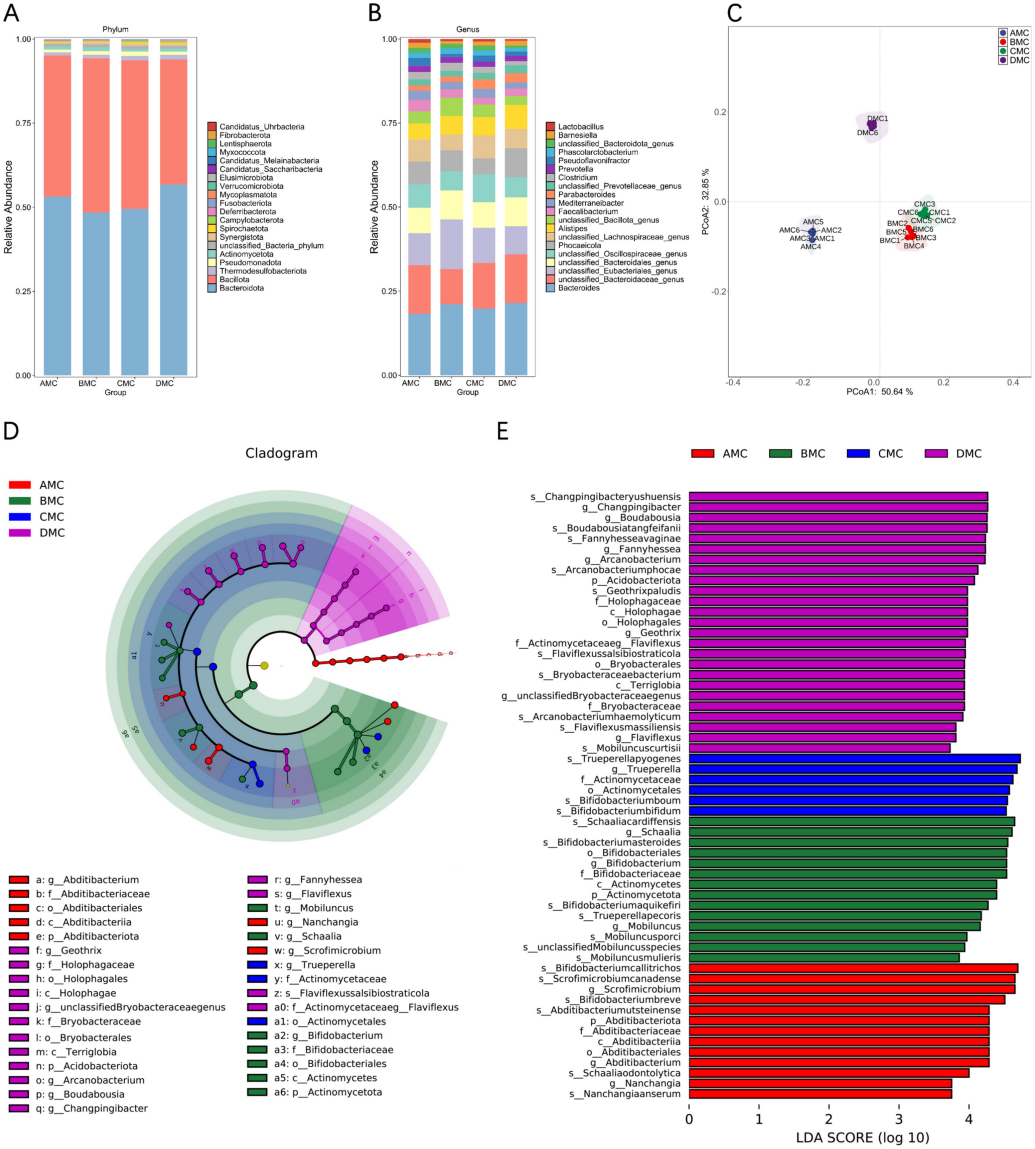
Microbial structure of the cecum under different proportions of fermented *Cordyceps militaris* mushroom substrate replacement diets. **(A,B)** Microbial compositions at the phylum level. **(C)** PCoA analysis. **(D,E)** LEfSe analyses.

In this current investigation, we compiled a grand total of 158 gigabytes of Illumina sequencing data from the 24 samples we gathered. After running a rigorous quality control process, we were left with a robust one billion reads, boasting an impressive average length of 1482.62 base pairs (refer to Supplementary Tables S2, S3 for more details). Our principal coordinate analysis (PCoA) yielded some fascinating insights, pinpointing considerable differences in the cecal microbiome of hens fed varying concentrations of fermented CMMS. Check out Figure 2C for the full story. In terms of microbial species composition, 20 high-ranking bacterial phyla and genera were selected for analysis based on abundance. At the phylum level, Bacteroidota and Bacillota were the dominant phyla, with relative abundances greater than 1%. The relative abundance of Bacteroidota was 53.13% for AMC, 48.41% for BMC, 49.51% for CMC, and 56.78% for DMC. The relative abundance of Bacillota was 41.92% in AMC, 45.83% in BMC, 44.11% in CMC, and 37.05% in DMC.

Moreover, at the phylum level, Thermodesulfobacteriota thrived more notably in the DMC and CMC batches, standing out against the other test groups (*p* < 0.05). The unclassified Bacteria phylum also showed a marked upsurge in the BMC batch, outpacing the rest of the groups (*p* < 0.05), while Spirochaetota boasts a notably higher presence in the DMC and CMC groups versus the others (*p* < 0.05). Furthermore, Mycoplasmatota was more prevalent in the BMC group than in the others (*p* < 0.05), and Verrucomicrobiota was especially abundant in the CMC group compared to the rest (*p* < 0.05; see Figure 2A). When zooming in on the genus level, the big players were Bacteroides, Phocaeicola, Alistipes, Faecalibacterium, Mediterraneiibacter, Clostridium, Prevotella, Parabacteroides, and Phascolarctobacterium, all boasting a relative abundance of over 1%.

In terms of the genus, *Bacteroides* was far more common in the BMC and DMC groups, showing a substantial uptick when compared to the rest of the test groups (the figure is below 0.05 significance). The relative frequencies of *Phocaeicola* and *Alistipes* were notably greater in the DMC group as opposed to the rest of the groups tested (each with a figure well below 0.05). A similar pattern was observed for *Faecalibacterium* and *Pseudoflavonifractor*, with their relative frequency on the up in the AMC group, distinctly outpacing the other test groups (both had figures well below 0.05). *Clostridium* and *Prevotella* also scored higher in the AMC and BMC groups relative to the other groups, with *p*-values under 0.05 for both. *Parabacteroides* was particularly prevalent in the DMC and CMC groups versus the rest of the experiment, while *Phascolarctobacterium* seemed to prefer the BMC group over the others, also with a *p*-value below 0.05 (refer to Figure 2B for details).

**Figure 3:**
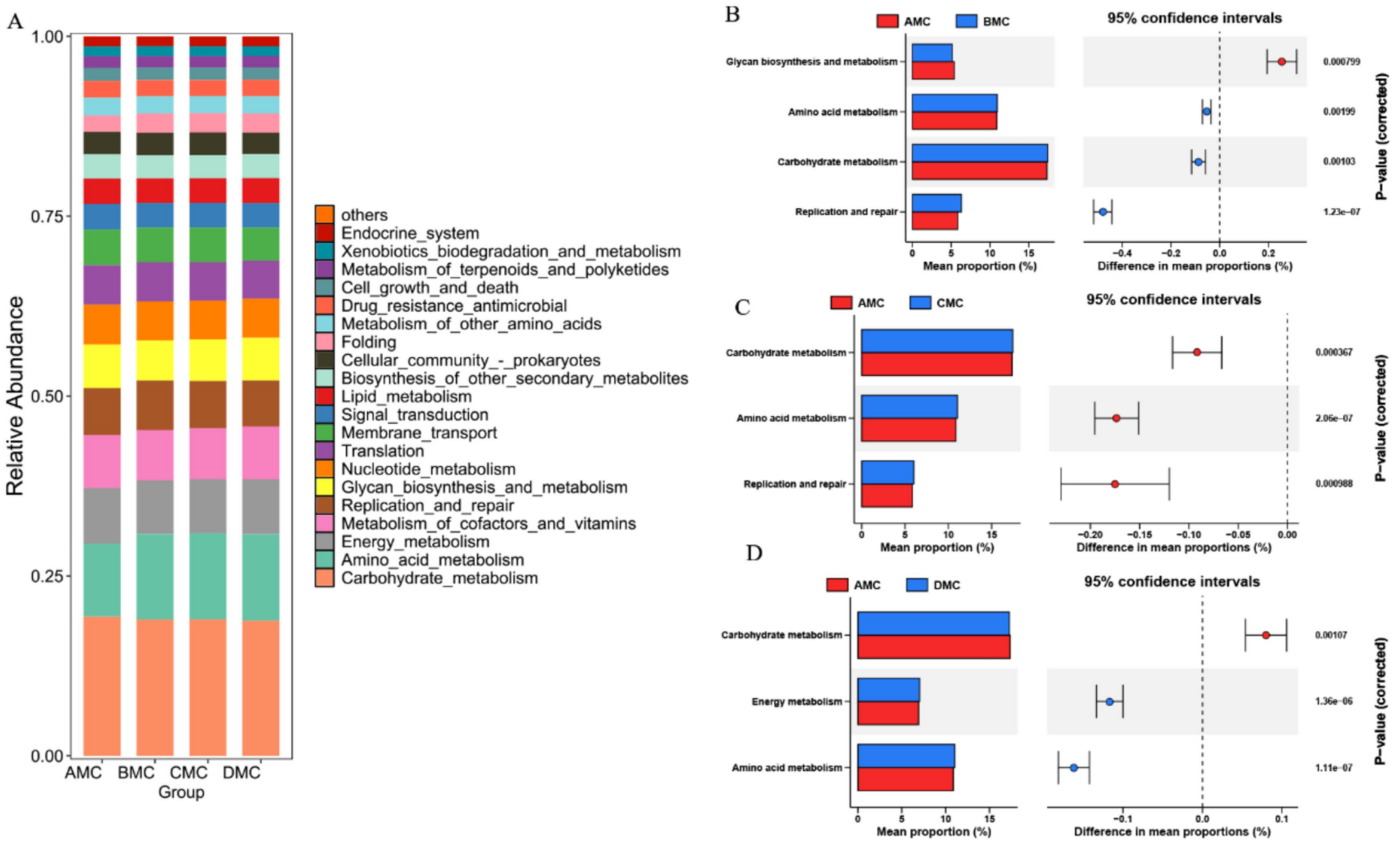
KEGG level 2 pathway analysis. **(A)** The 20 high-ranking KEGG level 2 pathways were ranked by relative abundance. **(B–D)** Stamp difference analysis.

LEfSe analysis revealed biomarkers in the cecum of laying hens fed diets supplemented with fermented CMMS at different ratios (Figures 2D,E). Additionally, the 20 high-ranking pathways by relative abundance in KEGG (level 2 pathways) were selected for analysis (Figure 3A). Stamp analysis revealed increases in glycan biosynthesis and metabolism, carbohydrate metabolism, amino acid metabolism, and replication and repair in the AMC group. Increases in amino acid metabolism, carbohydrate metabolism, and replication and repair were also detected in the BMC group. Finally, increases in energy metabolism and amino acid metabolism were also observed in the DMC group (Figures 3B–D).

### Changes in QS genes of microbial communities in the cecum of laying hens fed different proportions of CMMS

**Figure 4:**
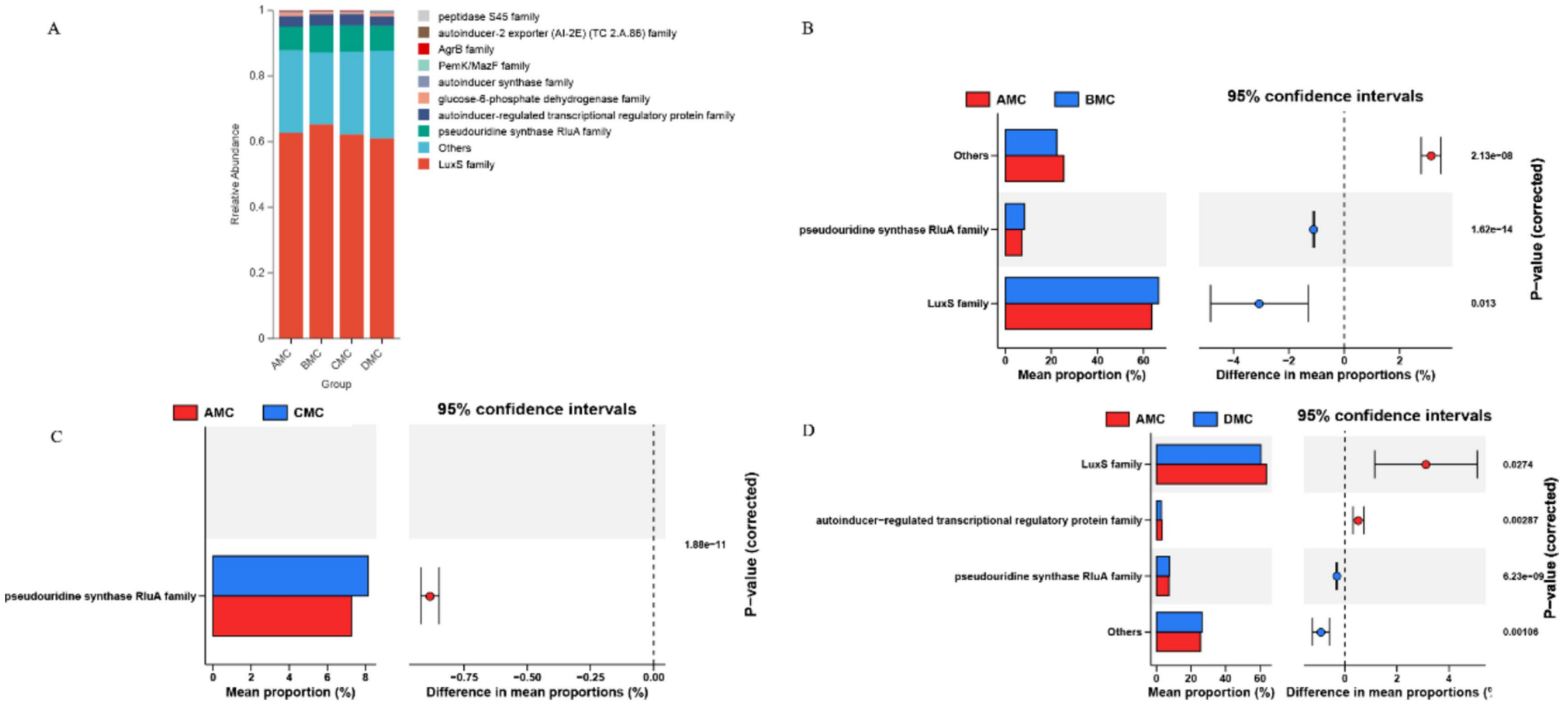
Analysis of QS function. **(A)** Relative abundance of QS genes. **(B–D)** Stamp difference analysis.

Nine QS genes were annotated in the cecal microbiota of laying hens. The relative abundance ranking of QS genes is as follows: LuxS family, pseudouridine synthase RluA family, autoinducer-regulated transcriptional regulatory protein family, glucose-6-phosphate dehydrogenase family, autoinducer synthase family, PemK/MazF family, AgrB family, autoinducer-2 exporter (AI-2E) (TC 2.A.86) family, and peptidase S45 family (Figure 4A). Stamp analysis revealed an increase in the pseudouridine synthase RluA family, LuxS family, and autoinducer-regulated transcriptional regulatory protein family in the AMC group. In the BMC group, there was an increase in the pseudouridine synthase RluA family and the LuxS family. Additionally, in the DMC group, the pseudouridine synthase RluA family showed an increase (Figures 4B–D).

### Changes in differential metabolites in laying hens fed different proportions of CMMS

**Figure 5:**
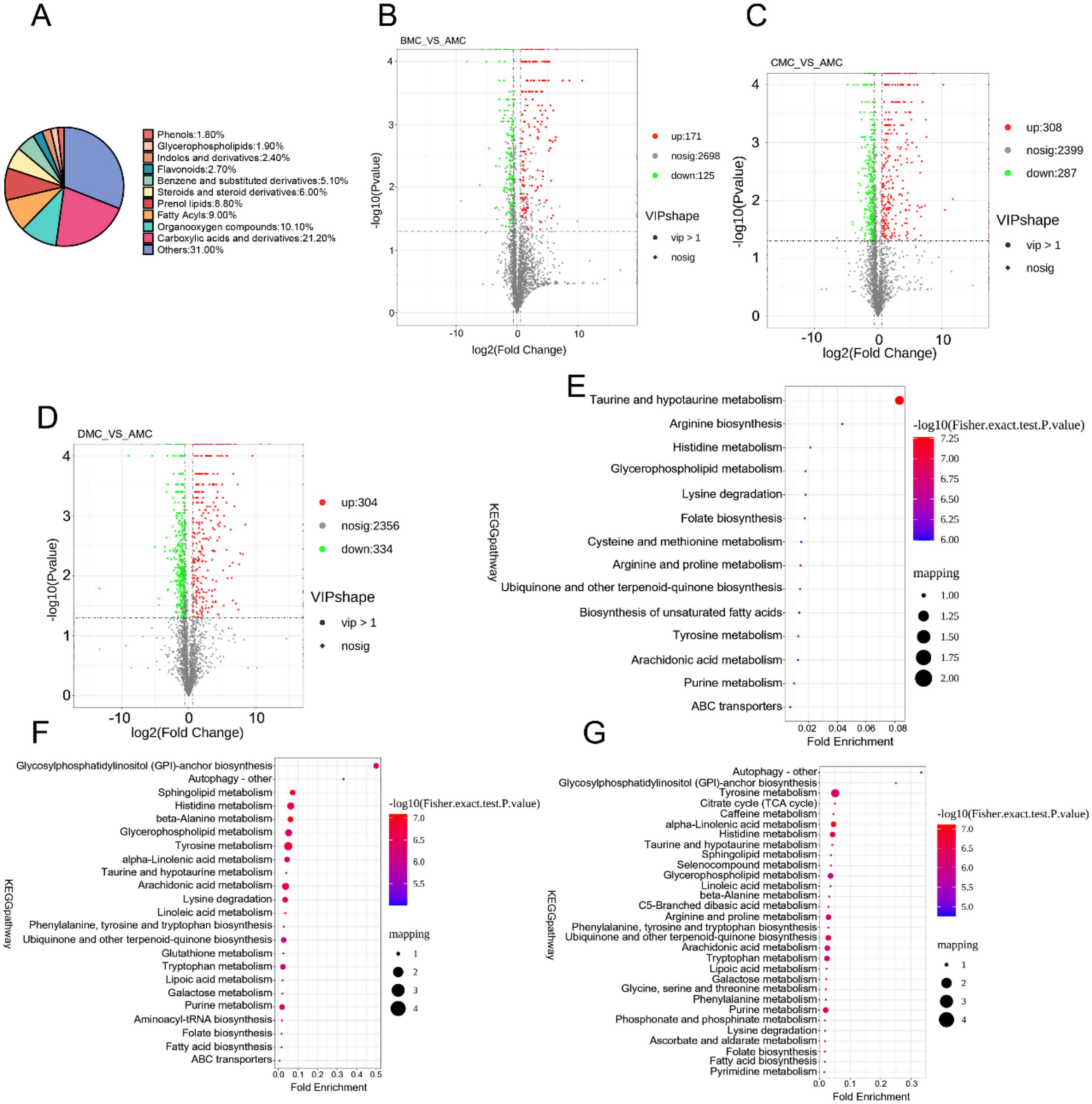
Metabolomics analysis. **(A)** Composition of differentially expressed metabolites. **(B–D)** Volcano plot analysis of differentially expressed metabolites. B: BMC_VS_AMC. C. CMC_VS_AMC. D. DMC_VS_AMC. **(E–G)** KEGG enrichment analysis of differentially expressed metabolites. **(E)** BMC_VS_AMC. **(F)** CMC_VS_AMC. **(G)** DMC_VS_AMC.

A nontargeted metabolomic analysis was conducted on the cecal chyme of laying hens in this study. A total of 1,529 differential metabolites were identified, ranked as follows: carboxylic acids and derivatives (21.20%), organooxygen compounds (10.10%), fatty acyls (9.00%), prenol lipids (8.80%), steroids and steroid derivatives (6.00%), benzene and substituted derivatives (5.10%), flavonoids (2.70%), indoles and derivatives (2.40%), glycerophospholipids (1.90%), phenols (1.80%), and others (31.00%) (Figure 5A). Volcano plot analysis of differentially expressed metabolites between samples revealed that BMC_VS_AMC had 171 upregulated and 125 downregulated differentially expressed metabolites (Figure 5B). For the CMC_VS_AMC comparison group, a total of 308 differentially expressed metabolites were found to be upregulated, while 287 differentially expressed metabolites were downregulated (Figure 5C). In the DMC_VS_AMC group, 304 differentially expressed metabolites exhibited upregulation, and 334 differentially expressed metabolites showed downregulation (Figure 5D). Furthermore, KEGG functional enrichment analysis was performed on these differentially expressed metabolites. The results indicated that the differentially expressed metabolites in the BMC_VS_AMC group were most significantly enriched in the taurine and hypotaurine metabolism pathway (Figure 5E). For the CMC_VS_AMC group, the differentially expressed metabolites were predominantly enriched in glycosylphosphatidylinositol (GPI)-anchor biosynthesis (Figure 5F), whereas the differentially expressed metabolites in the DMC_VS_AMC group were most highly enriched in the autophagy−other pathway (Figure 5G).

### Interactions between microorganisms and differential metabolites and QS genes

**Figure 6:**
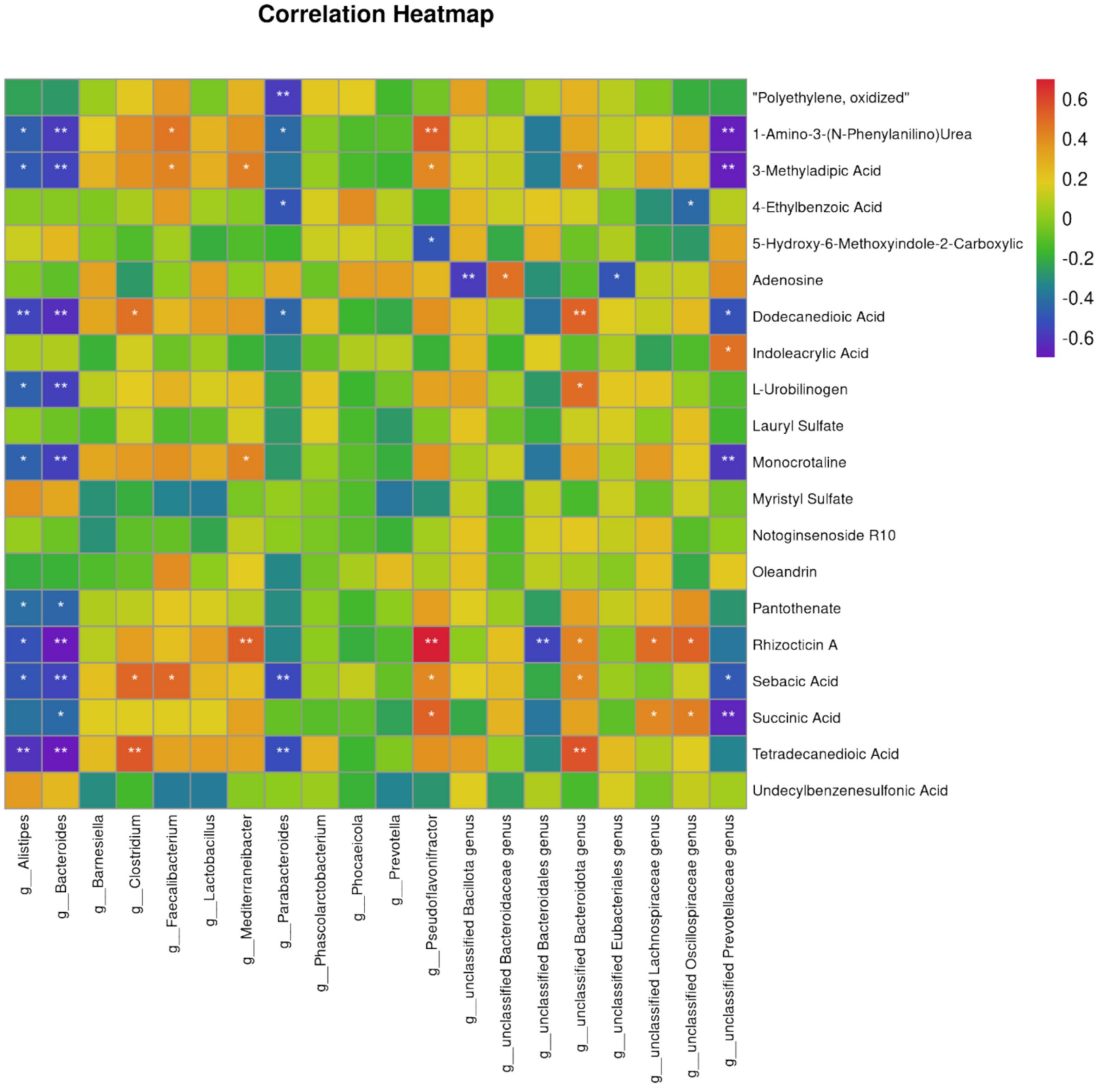
Correlation analysis between the microbial community and metabolites in the cecum. * *p* < 0.05, ** *p* < 0.01.

**Figure 7:**
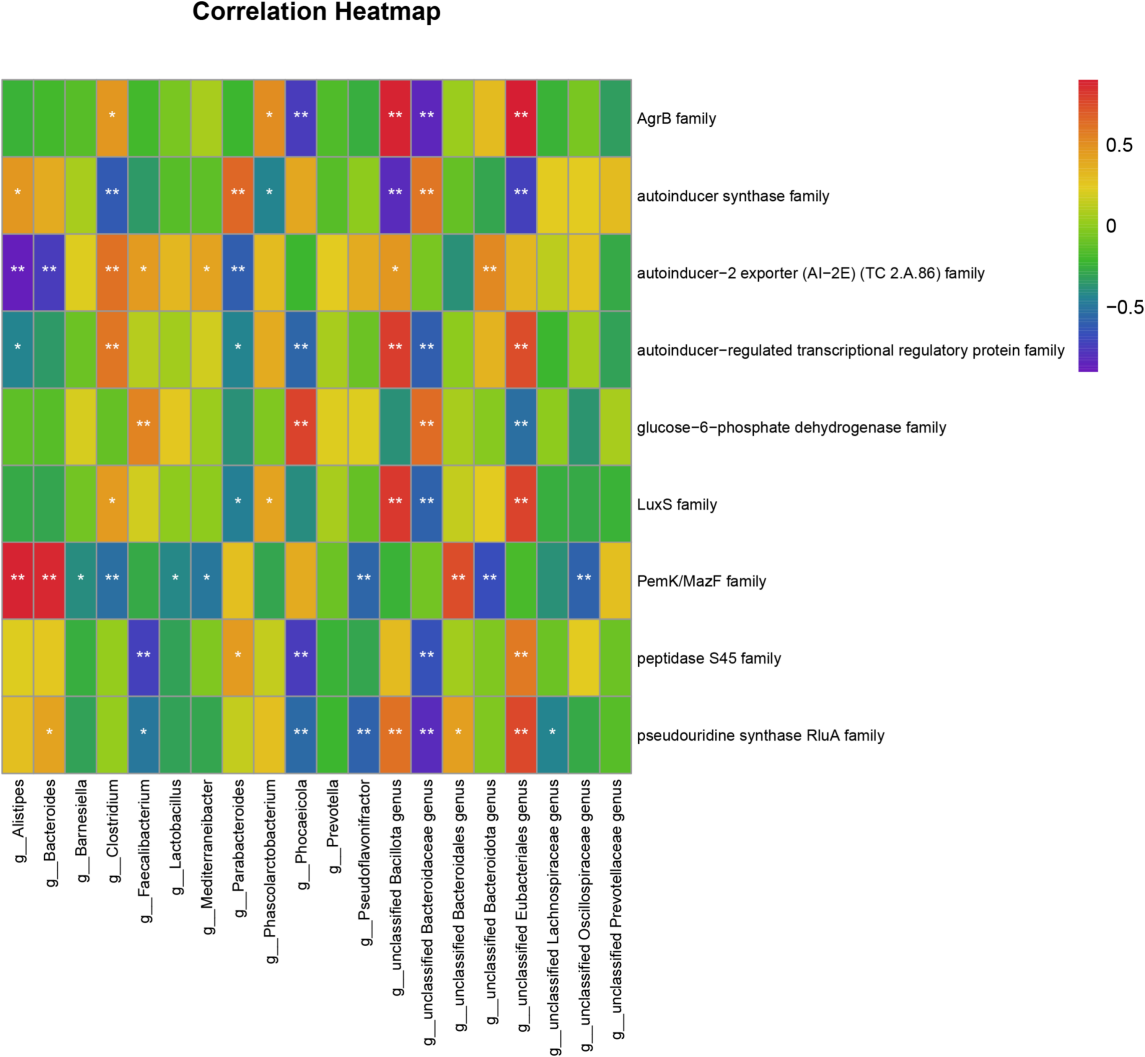
Correlation analysis between the microbial community of the cecum and quorum-sensing genes. * *p* < 0.05, ** *p* < 0.01.

Spearman correlation analysis was performed in this study between the 20 high-ranking bacterial genera, ranked by relative abundance, and the 20 high-ranking differentially expressed metabolites (also ranked by relative abundance) and QS genes. Correlation analysis of the top 20 differential metabolites (ranked by relative abundance) demonstrated the following relationships: *Alistipes* was significantly negatively correlated with dodecanedioic acid and tetradecanedioic acid (*r* < 0, *p* < 0.001); Analysis of microbial relationships revealed that Bacteroides had a striking inverse relationship with both tetradecanedioic acid and rhizocticin A (*r* < 0, *p* < 0.001), while Clostridium showed the opposite pattern, demonstrating a strong positive correlation with tetradecanedioic acid (*r* > 0, *p* < 0.001). Mediterraneibacter also jumped on the positive correlation bandwagon with rhizocticin A (*r* > 0, *p* < 0.001). Meanwhile, Parabacteroides was found to be in opposite territory, showing significant negative correlations with both tetradecanedioic acid and sebacic acid (*r* < 0, *p* < 0.001), whereas Pseudoflavonifractor was clearly on the same page with rhizocticin A and 1-amino-3-(N-phenylanilino) urea (*r* > 0, *p* < 0.001) (Figure 6). Diving deeper into quorum-sensing (QS) genes, we found that *Alistipes* was tight with the PemK/MazF family (*r* > 0, *p* < 0.001) but kept its distance from the autoinducer-2 exporter (AI-2E) family (TC 2.A.86) (*r* < 0, *p* < 0.001). *Bacteroides* was singing from the same hymn sheet, positively correlating with PemK/MazF (*r* > 0, *p* < 0.001) and negatively with AI-2E (*r* < 0, *p* < 0.001). On the other hand, *Clostridium* was playing by different rules, showing negative correlations with both the PemK/MazF family and autoinducer synthase family (*r* < 0, *p* < 0.001), while embracing the autoinducer-2 exporter (AI-2E) family and autoinducer-regulated transcriptional regulatory protein family with positive correlations (*r* > 0, *p* < 0.001). *Faecalibacterium* was out of step with the peptidase S45 family (*r* < 0, *p* < 0.001) but in harmony with the glucose-6-phosphate dehydrogenase family (*r* > 0, *p* < 0.001). *Parabacteroides* kept its distance from the autoinducer-2 exporter (AI-2E) family (*r* < 0, *p* < 0.001) but found common ground with the autoinducer synthase family (*r* > 0, *p* < 0.001). *Phocaeicola* was out of sync with the pseudouridine synthase RluA family, peptidase S45 family, autoinducer-regulated transcriptional regulatory protein family, and AgrB family (*r* < 0, *p* < 0.001), but in lockstep with the glucose-6-phosphate dehydrogenase family (*r* > 0, *p* < 0.001). Pseudoflavonifractor, for its part, was not seeing eye to eye with the pseudouridine synthase RluA family and the PemK/MazF family (*r* < 0, *p* < 0.001) (Figure 7).

## Discussion

Mounting evidence has shed light on how crucial gut microbiota is for maintaining overall host health. Changes in the intestinal microbial composition can significantly influence both physical condition and disease progression, according to numerous investigations (Li et al., 2023; Cao et al., 2023). Consequently, Therefore, the addition of feed additives to the diet and other health interventions have become common strategies for modulating the gut microbiota and ensuring optimal growth and health in animals (Maki et al., 2019; Zhou and Shen, 2026). With the rapid development of animal husbandry, competition between humans and livestock for grain has become a significant challenge to the advancement of animal husbandry (Schader et al., 2015). CMMS, as an unconventional feed, has a certain role in alleviating the conflict between humans and livestock over grain. Consequently, this investigation focused on commercial laying hens as the experimental subjects, thoroughly examining their capacity for feed efficiency using a range of approaches including production performance metrics, metagenomic analysis, and metabolomic profiling. The research sought to establish a solid theoretical framework that would inform the future application of CMMS in improving feed utilization within the laying hen population.

The findings of this research reveal that subjecting CMMS to fermentation caused a substantial transformation in its nutritional makeup, which in turn boosted the productivity, egg quality, and digestibility of laying hens. Additionally, it promoted digestive enzyme activity and increased serum immunoglobulin content. Studies have shown that a CM waste culture medium may offer potential benefits in improving egg quality and producing eggs with low cholesterol levels when used as a feed additive (Wang et al., 2015). Fermented enoki mushroom bran can enhance egg quality and inhibit the proliferation of gastrointestinal pathogens (Lee et al., 2014). Additionally, feeding oyster mushrooms to laying hens improves apparent digestibility and egg quality (Mahfuz et al., 2018). Our findings are consistent with the above reports. It has been demonstrated that fermented edible fungi or their culture medium have certain application potential as unconventional feed in laying hens. The specific feeding effects vary depending on the fermentation method, the source of the culture medium, and the type of edible fungi.

This research delved into the alterations in the gut microbiome of laying hens following the supplementation of fermented CMMS, utilizing metagenomic analysis to uncover the community structure and identifying various functionalities associated with it. The results showed that the dominant bacterial groups at the phylum level were Bacteroidota and Bacillota, with relative abundances of 53.13 and 41.92% in the AMC group, 48.41 and 45.83% in the BMC group, CMC (49.51, 44.11%), and DMC (56.78, 37.05%), respectively. These results align with earlier investigations into the gut microbial composition of poultry (Pandit et al., 2018; Ma et al., 2016). The Bacteroidota phylum plays a pivotal role in avian digestive processes and metabolic functions, such as breaking down complex carbohydrates (particularly cellulose and hemicellulose), generating short-chain fatty acids, and influencing overall host well-being and immune responses (Li W. et al., 2025; Limede et al., 2025; Akram et al., 2025; Jiang et al., 2025). Bacillota are crucial in animal nutrition, metabolism, energy supply, intestinal development, and immune regulation (Gardiner et al., 2020; Tardiolo et al., 2025; Indiani et al., 2018; Ouwehand et al., 2002). It has been demonstrated that fermented CMMS aids in promoting digestion, metabolism, and immune regulation in laying hens. In addition, our results on apparent digestibility and serum immunoglobulin also indicate that fermented CMMS promotes digestion and regulates immunity in laying hens.

At the genus level, *Bacteroides*, *Phocaeicola*, *Alistipes*, *Faecalibacterium*, *Mediterranibacter*, *Clostridium*, *Prevotella*, Parabacteroides, and *Phascolarctobacterium* are the dominant bacterial communities. *Bacteroides* in animals facilitate nutrient processing and uptake (Smith et al., 2006), maintaining intestinal homeostasis (Zafar and Saier, 2021), and influencing host health and immunity (Wexler, 2007). The findings reveal that *Bacteroides* levels were markedly higher in both the BMC and DMC treatment groups. A specific portion of the fermented CMMS appears to enhance nutrient digestion and absorption processes, as well as immune function in laying hens. Additionally, *Phocaeicola* plays a crucial role in preserving gut health and contributing to metabolic activities (Zhou et al., 2024). The findings revealed that *Phocaeicola* levels were markedly higher among the DMC subjects, hinting that fermented CMMS might give a leg up to gut balance and nutrient processing in laying hens. *Alistipes* has a variety of functions when it comes to animal physiology, wearing several hats across metabolic functions, immune responses, and therapeutic applications (Li et al., 2025a; Parker et al., 2020; Prosberg et al., 2016). The data revealed that *Alistipes* levels were considerably more prominent in the DMC cohort, pointing to the possibility that fermented CMMS could enhance the wellbeing of laying hens. When it comes to *Clostridium*, the story is complicated given its dual nature as both a friend and foe in animal health (Uzal et al., 2022). Notably, the AMC and BMC groups showed significantly greater *Clostridium* abundance compared to the rest of the experimental groups, which hints that fermented CMMS might bolster the disease resistance capabilities of these hens.

The KEGG functional annotation for the BMC group revealed a boost in amino acid and carbohydrate metabolism, as well as in replication and repair pathways. On the flip side, the DMC group experienced a surge in energy and amino acid metabolism. The functions of these components are consistent with the functional structure of the microbial community. This result suggests that fermented CMMS has potential as an unconventional feed application in egg chicken production. Additionally, it may have beneficial effects on nutrient digestion and absorption, as well as on maintaining intestinal homeostasis and enhancing disease resistance.

QS is a communication mechanism through which bacteria coordinate their behavior by secreting and sensing specific signaling molecules. When the number of bacteria reaches a certain density, the concentration of signaling molecules reaches a threshold, thereby activating a series of gene expressions that lead to synchronous changes in group behavior (Anburajan et al., 2023). The LuxS family genes encode an enzyme involved in the biosynthesis of AI-2 (Autoinducer-2). AI-2 is considered a QS signal molecule common to both gram-positive and gram-negative bacteria (Schauder et al., 2001). The quorum sensing system driven by AI-2 has been implicated in a range of bacterial physiological functions, such as biofilm development, the synthesis of virulence factors, antibiotic generation, and metabolic activities (Zhao et al., 2018). Interestingly, although no antibiotics were added to the diet of chickens, antibiotic resistance genes were annotated through metagenomic functional annotation (Shen et al., 2024). This may implied that bacteria can influence the production of antibiotics through QS pathways, which is an idea worth paying attention to and continuing to research. The research findings revealed a notable uptick in the LuxS family within the BMC cohort. This discovery suggests that fermented CMMS might play a significant part in modulating AI-2-induced bacterial quorum sensing. However, to pinpoint the exact mechanism at play, we require more comprehensive research. Pseudouridine synthase, the RluA family of pseudouridine synthases, is not a direct QS key protein (such as signal molecule receptors), but the QS system can regulate its gene expression. Therefore, if QS regulates enzymes of the RluA family, such regulation may indirectly influence the overall physiological state and metabolic activities of bacteria, and may even affect phenotypes related to collective behavior (Miyamoto and Meighen, 2006; Xu et al., 2025; Kalburge et al., 2017). Our findings indicate that the pseudouridine synthase RluA family is increased in the BMC and DMC groups, suggesting that fermented CMMS may have a potential impact on bacterial physiological status, metabolic activity, and phenotypes related to group behavior.

The gut microbiome influences host physiology via metabolite generation (Ren et al., 2018). Fatty acids are one of the most common carboxylic acids and are the primary form of energy storage in cells. Fatty acids are also components of phospholipids, sphingolipids, and triglycerides in cell membranes. Additionally, many intermediates in the tricarboxylic acid cycle are carboxylic acids (Hall and Zheng, 2011; Offermanns, 2014). This research reveals that among the varied metabolites with altered expression, carboxylic acids and their variants account for a robust 21.20%, making them the most prevalent. This result may suggest that fermenting CMMS at different proportions in the diet primarily affects energy metabolism and biofilm formation in laying hens. Organooxygen compounds are mainly involved in signal transduction, energy metabolism, and production, as well as antioxidant capacity, in living organisms (Hancock, 2021; Li et al., 2024; Wang et al., 2024; Wang et al., 2022). Our research results indicate that organooxygen compounds (10.10%) rank second among all differential metabolites. This suggests that the addition of fermented CMMS at varying proportions in the diet primarily affects signal transduction, energy metabolism, and antioxidant capacity in laying hens. Furthermore, the KEGG pathway enrichment analysis indicated that the metabolites that were found to be differentially expressed were predominantly clustered in three key pathways: the metabolism of taurine and hypotaurine, the biosynthesis of glycosylphosphatidylinositol (GPI) anchors, and autophagy-related processes. Taurine and hypotaurine, in particular, play a crucial role in the antioxidant game (Aruoma et al., 1988), bile acid binding (Russell, 2009), and osmotic regulation (Trachtman et al., 1988) pathways in animals. The biosynthesis of glycosylphosphatidylinositol (GPI)-anchor is essential for preserving cell membrane integrity, promoting intercellular communication, and governing the intricacies of cellular development (Desnoyer and Palanivelu, 2020). Autophagy–other roles in maintaining cellular homeostasis (Zhou et al., 2022) and immune regulation (Keller et al., 2023) in living organisms. This suggests that replacing different proportions of the diet with CMMS primarily affects antioxidant capacity, bile acid binding, maintenance of cellular homeostasis, and immune regulation in laying hens.

Additionally, there was a certain connection between microorganisms, their metabolites, and QS genes. This research suggests a notable inverse relationship between *Parabacteroides* and tetradecanedioic acid, as well as sebacic acid, with a correlation coefficient below zero and a statistically significant *p*-value less than 0.001. Conversely, *Pseudoflavonifractor* exhibited a significant direct correlation with rhizocticin A and 1-amino-3-(N-phenylalanino) urea, showing a correlation coefficient greater than zero and a *p*-value below 0.001. *Parabacteroides* promotes animal growth, improves digestive fermentation, and regulates immunity in animals (Feng et al., 2025; Zhang et al., 2025). Tetradecanedioic acid belongs to the class of dicarboxylic acids, which participate in host energy metabolism (Grego and Mingrone, 1995) and mediate host–microbial interactions, thereby affecting metabolic regulation (Lee et al., 2018). Sebacic acid participates in energy metabolism and regulates lipid metabolism (Liao et al., 2024). *Pseudoflavonifractor* is considered a beneficial bacterium (Kim et al., 2023), whose main end products are acetic acid and succinic acid (Sun et al., 2024), and which has immunomodulatory and metabolic regulatory effects (Tang et al., 2025). Rhizocticin A has antifungal properties (Jastrzębowska and Gabriel, 2015). This may suggest that the relative abundances of *Parabacteroides*, *Pseudoflavonifractor*, and other bacteria play a regulatory role in energy metabolism, lipid metabolism, and immune function, thereby exerting a positive influence on production performance. Of course, this is merely a correlation based on the data. Further validation and refinement are needed to determine the specific mechanisms involved. The study reveals a strong positive association between *Alistipes* and the PemK/MazF gene family (*r* > 0, *p* < 0.001). At the same time, *Faecalibacterium* is significantly negatively correlated with the peptidase S45 family (*r* < 0, *p* < 0.001). *Alistipes* regulate metabolism and affect intestinal immune function (Casto-Rebollo et al., 2023). The primary function of the PemK/MazF family is to regulate cell growth and metabolism, thereby enhancing bacterial survival rates. It is also linked to biofilm formation and the production of persistent cells (Qiu et al., 2022). *Faecalibacterium* can regulate intestinal homeostasis, improve intestinal barrier function, and reduce inflammation (Ferreira-Halder et al., 2017). The peptidase S45 family is involved in biological processes that require multistep enzymatic reactions or the coordination of multiple enzymes (Rawlings, 2013). This may suggest that there is a certain association between the relative abundances of *Alistipes*, *Faecalibacterium*, and other bacteria and certain quorum sensing genes, and that this association helps regulate intestinal homeostasis and improve intestinal barrier function, thereby positively impacting the production and health of laying hens. This interpretation is based on correlations observed in the data; further validation and refinement are needed to determine the specific mechanisms involved.

## Conclusion

In summary, this study determined the macroscopic and microscopic performance of laying hens following the replacement of their diet with fermented *Cordyceps militaris* mushroom substrate at different concentrations. The study characterized the production performance, apparent digestibility, microbial communities and their metabolites, and quorum sensing genes in laying hens under this substitution regimen. These changes enhanced energy metabolism and modulated the immune system in laying hens, providing insights into the hen’s microbiome and offering a reference for the feed utilization of fermented *Cordyceps militaris* mushroom substrate in laying hens.

## Data Availability

The data sets presented in this study can be found in the NCBI Sequence Read Archive (SRA) under accession numbers PRJNA1301039. The metabolomics data have been deposited to MetaboLights repository with the study identifier MTBLS12825.

## Footnotes

1
http://www.kegg.jp/kegg/compound/

2
http://www.kegg.jp/kegg/pathway.html

3
http://chemrich.fiehnlab.ucdavis.edu/
